# Factors Influencing the Quality of Life of Korean Elderly Women by Economic Status

**DOI:** 10.3390/ijerph17030888

**Published:** 2020-01-31

**Authors:** Myoungjin Kwon, Sun Ae Kim, Wi-Young So.

**Affiliations:** 1 Department of Nursing, Daejeon University, Daejeon 34520, Korea; mjkwon@dju.kr; 2 Department of Nursing, Korea National University of Transportation, Chungbuk 27909, Korea; 3 Sports and Health Care Major, College of Humanities and Arts, Korea National University of Transportation, Chungju-si 27469, Korea

**Keywords:** depressive symptoms, economic status, elderly, quality of life, women

## Abstract

*Purpose:* This study aimed to determine whether there are differences in the factors affecting the quality of life (QOL) of elderly women in South Korea according to their perceived economic status. *Methods:* Data were extracted from the Seventh Korea National Health and Nutrition Examination Survey conducted in 2016. The participants were 879 women over the age of 65 years, who were divided into three groups: high, medium, and low based on their perceived economic status. The study variables were classified into three categories: general characteristics, physical factors, and psychological factors. General characteristics included age, education level, employment, activity restriction, frequency of breakfast/week, frequency of lunch/week, and frequency of dinner/week. Physical factors included disease status, weight change, consumption of alcohol, number of days of walking per week, duration of walking at a time, body mass index (BMI), and discomfort due to changes in hearing. Psychological factors included stress, subjective body awareness, subjective health status, depressive symptoms, and QOL. Complex sample crosstabs and chi-square tests were conducted, and regression was performed to examine the association between the variables by economic status. *Results:* The factors that influenced the QOL of elderly women with low economic status were arthritis, alcohol consumption, subjective health status, and depressive symptoms, with an explanatory power of 54.3% (F = 14.94, *p* < 0.001). The factors that influenced the QOL of the medium economic status group were activity restriction, frequency of dinner/week, arthritis, number of days of walking per week, BMI, stress, subjective health status, and depressive symptoms, with an explanatory power of 48.6% (F = 9.82, *p* < 0.001). For the high economic status group, influential factors were age, restricted activity, arthritis, number of days of walking per week, stress, and depressive symptoms with an explanatory power of 49.0% (F = 69.76, *p* < 0.001). *Conclusions:* This study identified different factors that contributed to the QOL of elderly women by economic status.

## 1. Introduction

Both worldwide and in the Republic of Korea, the population of elderly individuals is increasing. In Europe, the proportion of people aged 80 and above is expected to double by 2080 compared to 2014. In Korea, the aging of the population is a critical situation that contributes to low fertility rates [1]. However, the researchers’ focus has shifted to health and the meaning of life rather than merely living longer. Moreover, the concept of “active aging” is becoming increasingly important, and determinants of active aging are being studied. Evidence suggests that active aging is determined not only by physical inactivity, cognitive impairment, and psychological disorders, such as depression, but also by economic factors such as income [2].

The World Health Organization (WHO) mentioned employment as one of the important factors for elderly adults to maintain an active life [3]. Moreover, interest in a healthy life is increasing as the lifespan of women is beginning to exceed that of men. In Korea, the life expectancy of elderly women is 85.7 years, which is higher than the 79.7 years for elderly men [4]. Thus, the ratio of women among the elderly population is expected to increase continuously, requiring systemic support and a focus on them. The number of senior citizens living alone in Korea has increased rapidly between 1960 and 2010, with a steep increase from 1% to 10% in men and 3% to 31% in women [5]. The proportion of elderly women living alone is very high compared to that of elderly men living alone, indicating that elderly women are particularly vulnerable. The difference in life expectancy between women and men is decreasing, but women still have a longer lifespan than do men, and may live longer in unhealthy circumstances [6]. Moreover, as medical technology advances, women have a higher average life expectancy with disabilities and an average life span without disability [7]. Additionally, senior citizens living alone exhibit lower health status and health promotion behaviors than those living with a spouse or other family members [8]. The health status of elderly adults is threatened by chronic diseases associated with aging [9]. Elderly women should be able to have goals and a purpose that is more than merely avoiding disease and decline but increasing their quality of life (QOL) by living well despite decreasing abilities [10]. Along with the rapid growth of the elderly population, their low economic status is a problem [11]. This is particularly true in the case of Korea, which is ranked very low, at 82, in terms of income security among 96 Organization for Economic Co-operation and Development countries and also has high rates of poverty and suicide [12]. These data suggest that elderly adults are at increased risk for economic and psychological challenges.

In addition, depression among the elderly individuals is a common problem though it does not always occur as a result of the aging process [13]. Depression among them is related to their social and personal situations, such as separation from a spouse by death, death of close friends, economic difficulty due to retirement, and chronic diseases [13]. Elderly persons face the double burden of adjustment to the novelty of old age while experiencing a sense of loss of the familiar as they attempt to adjust to changes in their financial situation [14].

The concept of QOL cannot be easily defined and has many dimensions [15,16]. Therefore, since various interacting variables affect it, the research on identifying and comparing the related variables is significant. Additionally, the dynamics of QOL change throughout the human lifespan [17]. This means that the current QOL may not remain the same until the end of life. Therefore, it is important to identify the age that needs social consideration and support during the changing QOL by using ethical and standardized procedures that do not harm the individuals being studied. However, to our knowledge, no previous study has identified the factors affecting QOL among elderly women based on their subjective perception of their economic status, although economic stability is an important factor affecting the lives of older adults [14]. Therefore, the purpose of this study was to examine the differences in general characteristics and physical and psychological factors of elderly women according to economic status and identify differences in factors affecting their QOL according to economic status.

## 2. Methods

### 2.1. Participants

Data for this study were extracted from the Seventh Korea National Health and Nutrition Examination Survey (KNHANES VII-1) conducted in 2016. A multi-stage clustered probability sampling was used. Raw data were collected using the stratified colonies system extraction method. This method can reduce biased sampling as much as possible. The data collection process was conducted under the supervision of Korea Centers for Disease Control and Prevention (KCDC), and the subjects who met the criteria of this study were extracted from the raw data. The present study included elderly women (over 65 years of age, weighted *n =* 879) who reported that their subjective perception of their economic status was low, medium, or high, which was drawn from the total sample (*N =* 7550). Missing data were excluded from the data analysis.

The KNHANES VII-1 was approved by the KCDC based on Korea Bioethics Law. All respondents voluntarily participated in the study and provided written informed consent prior to participation, and the institutional review board of KCDC provided approval (No. 2013-12EXP-03-5C).

### 2.2. Measurements

#### 2.2.1. Demographic Characteristics

The following general demographic characteristics of the participants were used from the survey data: age (65–74 years, ≥75 years), education level (≤elementary school, ≥middle school), employment (yes, no), restricted activity (yes, no), frequency of breakfast/week (≥5, ≤4), frequency of lunch/week (≥5, ≤4), and frequency of dinner/week (≥5, ≤4). Cutoff points were determined by the author according to the distribution of the participants’ characteristics.

#### 2.2.2. Physical Factors

The following data regarding physical factors of the participants were used from the survey: hypertension (yes, no), stroke (yes, no), coronary artery disease (yes, no), arthritis (yes, no), diabetes mellitus (yes, no), weight change (no change, loss, gain), consumption of alcohol (yes, no), number of days of walking per week (days/week: 0, 1–2, 3–6, 7), length of time walking (minutes: ≤29, 30–59, ≥60), body mass index (BMI; kg/m^2^, < 23, 23– < 25, ≥25) [18], discomfort due to changes in hearing (no discomfort, a little discomfort, a lot of discomfort).

#### 2.2.3. Psychological Factors

The following psychological data of the participants were used from the survey: stress (a lot, moderate, a little), subjective body awareness (thin, normal, overweight), subjective health status (good, normal, bad), depressive symptoms (score on Patient Health Questionnaire-9, PHQ-9), QOL (score on EuroQol-5 Dimension, EQ-5D). PHQ-9 was used to assess mental health at the primary health care center. EQ-5D and PHQ-9 were adjusted (validated) to the South Korean population [19,20]. This scale consists of 9 items each scored on a 4-point Likert scale from 0 to 3 points, with a total score ranging from 0 to 27 points. The higher the score, the greater the depressive symptoms. EQ-5D is a QOL indicator that assesses the five dimensions of exercise ability, self-care, daily life, pain/discomfort, depressive symptoms/anxiety. We used a weighted total score that combined individual scores on the five dimensions.

### 2.3. Statistical Analysis

Complex sample linear regression analysis procedures were used by considering sampling weights, stratification variables, and cluster variables using the IBM SPSS 25.0 program (IBM Corp., Armonk, NY, USA). The data used in this study was extracted using stratified cluster sampling method, and complex sample analysis method was used after correcting by applying weights when analyzing data according to the KCDC’s recommendation. Perceived economic status was determined from the relevant questions and classified into low, medium, and high levels. Cases with missing data were excluded during data analysis. Complex sample crosstabs and chi-square tests were conducted to compare the percentages or means of all the variables by economic status. Finally, variables that had a significant association in the bivariate analyses were included in a regression model. The significance level was set at 0.05.

## 3. Results

### 3.1. Comparison of General Characteristics of Participants According to Economic Status

As Table 1 indicates, there were significant differences among the economic status groups according to education level and employment (*p* < 0.05). The higher the economic status, the higher was the observed education level. Findings indicated that as economic level increased, the proportion of respondents who were employed also increased.

### 3.2. Comparison of Physical and Psychological Factors of Participants According to Economic Status

As Table 2 indicates, there were significant differences among the groups in alcohol use, number of days of walking per week, and duration of walking at a time, all of which are physical factors (*p <* 0.001). Alcohol consumption was found to be the highest among participants at the medium economic status (*p <* 0.05); number of days of walking per week was highest for the high economic status group (*p <* 0.001). In terms of the duration of walking at a time, more than 60 minutes and 30–59 minutes were more frequent for the low and high groups, respectively. There were differences in subjective health, depressive symptoms, and QOL among the groups (*p <* 0.05). Higher economic status was linked to higher subjective health; depressive symptoms increased in severity in the low and medium economic status groups, and QOL was the lowest in the low economic status group.

### 3.3. Factors Affecting QOL of Participants According to Economic Status

As Table 3 indicates, factors affecting QOL among participants with low economic status were the presence of arthritis, alcohol consumption, subjective health, and depressive symptoms, and the explanatory power of these factors was 54.3% (*F* = 14.94, *p* < 0.001). QOL was higher among participants without arthritis and those who did not drink, with better subjective health and lower depressive symptoms than those with arthritis and who used alcohol.

Factors affecting QOL among participants with medium economic status were restricted activity, frequency of dinner/week, the presence of arthritis, number of days of walking per week, BMI, stress, subjective health, and depressive symptoms, with an explanatory power of 48.6% (*F* = 9.82, *p* < 0.001). QOL was higher for participants without arthritis, those with BMI less than 25 kg/m^2^, and with higher subjective health status than the other participants. The QOL was lower among participants with activity restriction, more than 5 dinners per week, fewer days of walking per week, and higher stress and depressive symptoms.

Factors affecting QOL among participants in the high economic status group were age, activity restriction, presence of arthritis, number of days of walking per week, stress, and depressive symptoms, and their explanatory power was 49.0% (*F* = 69.76, *p* < 0.001). QOL was higher among participants who were younger and did not have arthritis. It was lower among participants with activity restriction, fewer days of walking per week, higher stress, and greater depressive symptoms than other participants.

## 4. Discussion

The study results revealed that there were differences in general characteristics according to differences in respondents’ perception of their economic status. Education and economic status are already known to be correlated in previous studies [21,22]. Additionally, a study aiming to identify the factors affecting QOL of 120 elderly participants with a mean age of 69 years reported that economic status is an important factor for QOL [2]. Moreover, a previous study has found that the higher the educational background, the higher is the level of employment and the lower the unemployment rate [23]. These results are consistent with the current research. In other words, elderly women with a higher education level can engage in high wage work and have a higher chance of being regular permanent workers than non-regular workers. This allows them to remain employed for longer time in life as regular job is related to continuous job. Further, elderly women with low income may have poor health access due to their low accessibility to medical services and inability to manage their health [24].

In addition, there were differences in economic status based on subjective health status, depressive symptoms, and QOL among elderly adults. A previous study showed that the employment of the elderly is a more fundamental factor than a change in their economic position [25], which is consistent with the present study’s results. The QOL of elderly women who indicated low economic status was lower in those who reported having arthritis, consumed alcohol, rated their subjective health as poor, and scored higher on depressive symptoms.

In this study, depressive symptoms were found to be a critical factor of QOL regardless of economic status among elderly women, suggesting that it should be carefully dealt with regardless of economic status. Losing a significant person or job due to retirement are inevitable life events for elderly adults, and these losses can lead to feelings of loneliness and isolation, resulting in depressive symptoms [26]. Previous research has found that depressive symptoms vary by sex and are twice as high among women as that among men [27]. Thus, elderly women are highly vulnerable to depressive symptoms. During the transition from loneliness to depression, social support has been reported as a protective factor against the progression of depression [28]. Sociocultural variables, along with social support, can alleviate depression [29], suggesting that efforts to control the depression of elderly women through intervention programs based on social support and sociocultural variables are necessary.

Moreover, economic status directly and indirectly affects QOL and subjective well-being [21]. Future research should a conduct path analysis of depressive symptoms among elderly women with economic status as an intermediary cause. A more specific correlational analysis or factor analysis is necessary as studies that use objective indices such as actual asset levels are required. This study also found differences in depressive symptoms according to economic status with the expected result indicating that higher economic status was related to lower levels of depressive symptoms. However, no difference by economic status was found, as depressive symptoms were significant predictors of low QOL in all economic status groups. Having arthritis was associated with lower QOL, and arthritis, along with depressive symptoms, affected participants at all economic levels in this study. It has been reported that the loss of labor and productivity due to musculoskeletal disorders has cost 240 billion EUR. In addition, 67% of the respondents in that study reported a decrease in QOL due to pain from musculoskeletal disorders, and 49% reported limited work [30].

The findings of another study indicated that QOL of the elderly individuals suffering from musculoskeletal disease with an accompanying disease was lower, which is consistent with our findings [31]. Among chronic diseases affecting the elderly, arthritis showed the second highest prevalence (33.4%) after hypertension (56.7%), as it is a representative musculoskeletal disease along with backache and sciatic neuralgia (21.1%) and osteoporosis (14%) [32]. In particular, a decrease in mobility due to arthritis may affect the daily life of the elderly persons and impairments to activities of daily living is related to decreased QOL in them [33,34]. In addition, muscles become weakened due to decreased daily life function and mobility [35], which in turn exacerbates the decrease in daily life function. The current finding that decreased daily life function due to arthritis is a factor that decreases QOL is consistent with the findings of previous research on the relationship between the maintenance of independent daily life and QOL [36]. These previous results are consistent with the findings of this study. However, this study included only arthritis among chronic diseases without identifying other accompanying chronic diseases. Future studies should separately identify cases with musculoskeletal disease and those with other accompanying diseases.

Alcohol use was associated with QOL in study participants with low economic status, which differed from the results for participants in the other economic status groups. Elderly women in the low group who reported consuming alcohol exhibited lower QOL than those who did not drink alcohol. Alcohol use in a previous study was also found to be a factor significantly affecting QOL [37]. Another previous study reported that depression significantly lowers life satisfaction and drinking alcohol is a parameter that had a negative impact on life satisfaction [38]. Elderly women who perceived themselves as having low economic status regarded their economic level as threatening their QOL, whereas elderly women with medium or high economic status were able to view their economic status as an unimportant issue. Alcohol consumption has diverse patterns, making it difficult to compare the results identically [39]. Many advanced studies have reported the relationship between health-related QOL and consumption of alcohol [40,41]. This is assumed that narrow based report about alcohol consumption makes it handle the health-related quality of life with regard to health issue from over-consumption of alcohol rather than relationship with overall quality of life. Nevertheless, problems from excessive alcohol consumption in Korea are continuously increasing [42]. Thus, a replication study that examines alcohol use and QOL in the elderly population is needed to clarify these conflicting results.

Additionally, participants with medium and high economic status exhibited lower QOL if their activities were restricted than did those who faced no such restriction. However, restricted activity was not a significant variable for participants with low economic status. These results might be explained by the fact that participants with low economic status had a high need for employment despite having arthritis; therefore, they could not recognize any restriction on their activity as an important factor. While we could not find any previous research examining this association, we could link these results to the fact that economic gaps are related to health disparities. One study reported that access to medical care was restricted by economic status and that older adults with higher economic status exhibited better health behavior [43]. However, this study has certain limitations. As the data of this study were controlled by KCDC and the Korean government, it was impossible to modify variables. In the future, we will seriously consider another research method. In addition, when planning individual research in the future, it is necessary to design research that can lower the bias of data and improve its usability. This study found that there was difference in factors that affect QOL according to economic status in female elderly adults. Thus, more in-depth study of female elderly individuals should be conducted.

## 5. Conclusions

This was a population-based study to determine whether there are differences in factors affecting QOL according to perceived economic status of elderly Korean women. The data were collected from the KNHANES VII-1. The respondents were elderly women aged over 65 years who reported their economic status. The study results revealed differences in the factors influencing QOL according to the perceived economic status of elderly women. This suggests that it is necessary to change how we look at elderly women who have the same characteristics of economic and social vulnerabilities. Specific approaches and intervention strategies to improve the QOL of elderly women with different economic statuses need to be devised. This study is meaningful as it offers evidence for formulating new strategies to provide efficient and high-quality care for elderly women who currently represent a large proportion of the population and are expected to increase further in the future. Based on the results of this study, it is necessary to differentiate strategies according to the perceived economic status in order to implement education, promotion, and mediation programs to improve the QOL of elderly women. In addition, this research was based on the perceived economic status of elderly women, that is, subjective economic status. Furthermore, it is necessary to explore the factors affecting the QOL according to the actual income or earnings, salary, or GDP of elderly women as well as men.

## Figures and Tables

**Table 1:** Demographic Characteristics by Economic Group Membership.

Characteristics		Low (*n* = 220)	Medium (*n* = 438)	High (*n* = 221)	χ^2^ (*p*)
		*n* (weighted %)	*n* (weighted %)	*n* (weighted %)	
Age (years)	65–74	130 (55.0)	259 (55.2)	128 (50.2)	1.660 (0.547)
	≥ 75	90 (45.0)	179 (44.8)	93 (49.8)	
Education Level	≤ Elementary School	173 (84.5)	306 (73.7)	133 (62.3)	28.264 (<0.001)
	≥ Middle School	31 (15.5)	102 (26.3)	77 (37.7)	
Employment	Yes	38 (16.3)	115 (26.5)	46 (25.1)	9.336 (0.015)
	No	173 (83.7)	294 (73.5)	158 (74.9)	
Restricted Activity	Yes	41 (19.1)	101 (23.3)	45 (20.0)	1.809 (0.461)
	No	171 (80.9)	318 (76.7)	161 (80.0)	
Frequency of Breakfast/Week	≥ 5	172 (90.0)	357 (90.9)	172 (92.4)	0.771 (0.775)
	≤ 4	21 (10.0)	26 (9.1)	13 (7.6)	
Frequency of Lunch/Week	≥ 5	176 (91.3)	340 (88.8)	176 (93.3)	3.413 (0.243)
	≤ 4	17 (8.7)	43 (11.2)	139 (6.7)	
Frequency of Dinner/Week	≥ 5	179 (93.9)	363 (94.6)	179 (93.3)	0.412 (0.848)
	≤ 4	14 (6.1)	20 (5.4)	10 (6.7)	

**Table 2:** Physical and Psychological Characteristics of the Groups.

Characteristics			Low (*n* = 220)	Medium (*n* = 438)	High (*n* = 221)	χ^2^ (*p*)/*p*
			*n* (weighted %)	*n* (weighted %)	*n* (weighted %)	
Physical Factors	Hypertension	Yes	114 (53.2)	267 (60.8)	120 (55.0)	4.141 (0.224)
		No	107 (46.8)	169 (39.2)	100 (45.0)	
	Stroke	Yes	10 (4.7)	22 (4.7)	7 (3.9)	0.270 (0.895)
		No	198 (95.3)	398 (95.3)	205 (96.1)	
	Coronary artery disease	Yes	15 (5.8)	30 (7.3)	14 (6.9)	0.496 (0.796)
		No	193 (94.2)	390 (92.7)	198 (93.1)	
	Arthritis	Yes	101 (48.5)	185 (44.0)	98 (43.6)	1.370 (0.593)
		No	105 (51.5)	235 (56.0)	114 (56.4)	
	Diabetes Mellitus	Yes	63 (29.1)	103 (23.7)	46 (22.5)	3.099 (0.334)
		No	158 (70.9)	333 (76.3)	174 (77.5)	
	Weight Change	No change	153 (71.7)	298 (70.7)	168 (76.9)	3.192 (0.656)
		Loss	32 (15.0)	68 (16.1)	28 (12.7)	
		Gain	26 (13.3)	56 (13.2)	22 (10.4)	
	Alcohol consumption	Yes	115 (55.4)	257 (61.3)	123 (56.2)	13.189 (0.049)
		No	96 (44.6)	166 (38.7)	94 (43.8)	
	Number of days of walking per week (day/week)	0	91 (41.9)	128 (31.0)	41 (17.3)	37.031 (<0.001)
		1–2	28 (12.9)	61 (14.2)	34 (15.9)	
		3–6	47 (23.2)	111 (24.3)	74 (35.9)	
		7	38 (22.0)	111 (30.5)	60 (30.9)	
	Length of time walking at a time (min)	≤ 29	64 (30.1)	159 (34.8)	84 (38.4)	34.859 (<0.001)
		30–59	49 (23.8)	124 (29.0)	84 (40.4)	
		≥ 60	108 (46.1)	154 (36.1)	52 (21.2)	
	BMI (kg/m^2^)	≤ 22.9	81 (37.9)	133 (30.5)	67 (28.9)	7.093 (0.274)
		23–24.9	42 (18.8)	107 (25.7)	57 (26.7)	
		≥ 25	91 (43.3)	189 (43.8)	94 (44.4)	
	Discomfort due to Hearing Changes	No discomfort	140 (63.3)	289 (67.7)	154 (74.2)	10.799 (0.143)
		A little discomfort	49 (23.1)	105 (23.5)	42 (16.0)	
		A lot of discomfort	31 (13.3)	38 (8.4)	19 (9.0)	
Psychological Factors	Stress	Feel a lot	59 (23.9)	109 (25.7)	45 (19.5)	4.029 (0.569)
		Moderate	87 (43.2)	192 (45.4)	105 (47.4)	
		Feel a little	64 (32.9)	117 (28.9)	68 (33.1)	
	Subjective Body Awareness	Thin	50 (23.4)	66 (16.1)	33 (16.2)	8.451 (0.173)
		Normal	89 (42.5)	170 (40.0)	96 (44.1)	
		Overweight	72 (34.1)	186 (43.9)	88 (39.7)	
	Subjective Health Status	Good	28 (15.0)	62 (15.6)	42 (21.1)	18.067 (0.009)
		Normal	82 (37.9)	179 (41.5)	115 (49.9)	
		Bad	97 (47.1)	179 (42.9)	55 (29.0)	
	Depressive symptoms (score)		4.890	3.840	2.610	<0.001
	Quality of Life (score)		0.800	0.850	0.880	0.049

Results are expressed as complex sample mean or n (%). Results of chi-square tests are provided.

**Table 3:** Linear Multiple Regression of Factors Influencing Quality of Life by Economic Group.

Model			*β*	*SE*	*t*	*p*	*R*²	*F*	*p*
Low Economic Status	Arthritis	Yes(ref)	1.000				0.543	14.946	<0.001
		No	0.071	0.022	3.170	0.002			
	Alcohol consumption	Yes(ref)	1.000						
		No	0.055	0.020	2.765	0.006			
	Subjective health status	Good	0.110	0.039	2.842	0.005			
		Normal	0.134	0.025	5.313	<0.001			
		Bad(ref)	1.000						
	Depressive symptoms		−0.007	0.003	−2.313	0.022			
Medium Economic Status	Activity restriction	Yes	−0.088	0.021	−4.118	<0.001	0.486	9.820	<0.001
		No(ref)	1.000						
	Frequency of dinner/week	≥ 5	−0.093	0.034	−2.688	0.008			
		≤ 4(ref)	1.000						
	Arthritis	Yes(ref)	1.000						
		No	0.041	0.015	2.661	0.009			
	Number of days of walking per week (day/week)	0	−0.099	0.020	−4.969	<0.001			
		1–2	−0.021	0.020	−1.037	0.301			
		3–6	−0.020	0.019	−1.060	0.291			
		7(ref)	1.000						
	Length of time walking at a time (min)	≤ 29	−0.047	0.016	−3.034	0.003			
		≥ 60(ref)	1.000						
	Body mass index (kg/m^2^)	≤ 22.9	0.044	0.024	1.794	0.075			
		23–24.9	0.040	0.020	2.048	0.042			
		≥ 25(ref)	1.000						
	Stress	Feel a lot(ref)	1.000						
		Moderate	−0.027	0.014	−1.927	0.056			
		Feel a little	−0.081	0.017	−4.877	<0.001			
	Subjective health status	Good(ref)	1.000						
		Normal	0.061	0.015	4.064	<0.001			
		Bad	0.084	0.021	4.002	<0.001			
	Depressive symptoms		−0.005	0.002	−2.414	0.017			
High Economic Status	Age (year)	65–74	0.075	0.020	3.782	<0.001	0.490	69.764	<0.001
		≥ 75(ref)	1.000						
	Activity restriction	Yes	−0.057	0.026	−2.153	0.033			
		No(ref)	1.000						
	Arthritis	Yes(ref)	1.000						
		No	0.086	0.022	3.851	<0.001			
	Number of days of walking per week (day/week)	0	−0.085	0.030	−2.863	0.005			
		1–2	−0.064	0.026	−2.498	0.014			
		3–6	−0.003	0.015	−0.202	0.840			
		7(ref)	1.000						
	Stress	Feel a lot(ref)	1.000						
		Moderate	−0.048	0.019	−2.562	0.011			
		Feel a little	−0.052	0.024	−2.206	0.029			
	Depressive symptoms		−0.005	0.002	−2.105	0.037			

Results of multiple regression analysis are provided.
